# Raman Spectroscopic Methods for Classification of Normal and Malignant Hypopharyngeal Tissues: An Exploratory Study

**DOI:** 10.4061/2011/632493

**Published:** 2011-07-24

**Authors:** Parul Pujary, K. Maheedhar, C. Murali Krishna, Kailesh Pujary

**Affiliations:** ^1^Department of Otorhinolaryngology and Head & Neck Surgery, Kasturba Medical College, Manipal University, Karnataka, Manipal 576 104, India; ^2^Department of Radiotherapy and Oncology, Kasturba Medical College and Center for Atomic and Molecular Physics, Manipal University, Karnataka, Manipal 576 104, India; ^3^Chilakapati Laboratory, Cancer Research Institute (CRI), Advanced Center for Treatment, Research and Education in Cancer (ACTREC), Tata Memorial Center (TMC), Kharghar, Navi Mumbai 410 210, India

## Abstract

Laryngeal cancer is more common in males. The present study is aimed at exploration of potential of conventional Raman spectroscopy in classifying normal from a malignant laryngopharyngeal tissue. We have recorded Raman spectra of twenty tissues (aryepiglottic fold) using an in-house built Raman setup. The spectral features of mean malignant spectrum suggests abundance proteins whereas spectral features of mean normal spectrum indicate redundancy of lipids. PCA was employed as discriminating algorithm. Both, unsupervised and supervised modes of analysis as well as match/mismatch “limit test” methodology yielded clear classification among tissue types. The findings of this study demonstrate the efficacy of conventional Raman spectroscopy in classification of normal and malignant laryngopharyngeal tissues. A rigorous evaluation of the models with development of suitable fibreoptic probe may enable real-time Raman spectroscopic diagnosis of laryngopharyngeal cancers in future.

## 1. Introduction

“Hypopharyngeal,” also known as “laryngopharyngeal,” cancers are tumors of a subsite of the upper aerodigestive tract within the group of head and neck malignancies. The hypopharynx is the region between the oropharynx and the esophageal inlet. Approximately 7% of all cancers of the upper aerodigestive tract are of hypopharyngeal origin [1]. Incidence of these cancers seems to be four to five times less common compared to laryngeal cancers. All pharyngeal subsites accounted for approximately 1,24,000 cancer cases worldwide in 2002 [1]. India has the second largest population in the world with predominant oral, pharyngeal, and oesophageal cancers among females and laryngeal cancers among males [2, 3]. This is attributed to intake of various tobacco products like “paan.” Smoked tobacco and slaked lime in paan are said to have synergistic carcinogenic effect in the upper aerodigestive tract [4]. Hypopharyngeal cancers are usually squamous cell carcinomas (SCCs) and are notorious as they usually present in advanced primary disease with or without nodal metastasis. The reconstruction after wide surgical resection in such cases is challenging and may increase morbidity and mortality. Hence early diagnosis is essential. A Contrast-enhanced computed tomography (CT) or a magnetic resonance imaging (MRI) of the head and neck is the mainstay initial radiological evaluation of these cancers [5]. PET scan is the latest imaging technique emerged to detect residual, recurrent tumors or secondaries. Due to occasional false positive results in cases of active inflammation or infection, this technique is also eventually dependant on biopsy for confirmation. Presently, rigid endoscopy and biopsy as mandatory as histopathology is the current gold standard for tissue diagnosis. The clinicians are dependant on skilled pathologist for accurate diagnosis. Moreover, the tissue sample may be inadequate or the pathologist may request deeper “repeat” multiple tissue biopsy. In anticipation of the biopsy report, patient may lose three to four days before active intervention of treatment. Only the gross manifestation of tissue changes arouse suspicion making assessment by hypopharyngoscopy under general anaesthesia mandatory. This subjects the patient to possibility of excess bleeding or anaesthesia-related complications especially in elderly patients and/or postoperative pain while swallowing. The tissue biopsy is especially challenging in irradiated cases wherein frank growth (residual or recurrent) may be obscured due to induration or Edema. The other modalities of tissue diagnosis may be particularly necessary as confirmation in false positive interpretation [6] of malignancies. Hence, it is crucial to depend on alternative methods to (1) confirm malignancy, (2) detect latent or early mitotic changes before gross appearance of abnormal tissue, and (3) extend its application to *in vivo* or *in situ* conditions.

Optical spectroscopic methods such as autofluorescence [7–9], Fourier transform infrared (FTIR) [10, 11], and Raman [12] have been the other methods of detection of malignancies. These optical methods attribute noninvasiveness unlike a painful biopsy with no prerequisite for staining or sample preprocessing. All are amenable to multivariate statistical tools for easy analysis.

Among the above-mentioned optical methods, fluorescence and FTIR are more popular due to simple instrumentation. Raman spectroscopy offers distinct advantages compared to other popular optical techniques. This is because less harmful near-infrared radiation is used for excitation with easy extraction of information due to distinct and sharp spectral features. The water content in tissues may not deter precision in diagnosis for *in vivo* and *in situ* future applications. 

The shortcoming of fluorescence technique is that it may require an experienced ENT specialist to detect laryngeal cancer *in vivo* and it has had low specificity in tissue diagnosis. The method of diagnosis by contact endoscopy for preoperative screening of laryngeal malignancy also has limitation in its application. It allows assessment of only the superficial layers of epithelium [13]. 

The mode of diagnosis by tissue analysis using Raman spectroscopy has been proved to be a useful tool in classifying oral [14–17], brain [18], breast [19–21], cervical [22, 23], ovarian [24], nasopharyngeal [25], laryngeal [26–28], gastrointestinal tract [29–33], and skin [34] malignancies. 

There are only three series of laryngeal and one study of nasopharyngeal cancers reported [25–28] so far. The significance of diagnosing hypopharyngeal cancers early is evident by the fact that these present worst prognosis especially because most of them present in advanced stages. With application of lasers in head and neck surgeries, a precise and optimum excision of a localized hypopharyngeal lesion is possible with good long-term prognosis. This highlights emphasis on early detection of hypopharyngeal tissue malignancy. Raman spectroscopy methodologies are ideal tools for noninvasive screening of population due to it's suitability for *in situ* and *in vivo* measurements. Since no spectroscopy study of hypopharyngeal cancers has been reported in the literature to date, we have carried out an exploratory conventional *ex vivo* Raman spectroscopy study of hypopharyngeal squamous cell carcinoma. We found that conventional Raman spectroscopy, unlike microscopy, probes larger areas thus provides representative spectra. Conventional Raman studies of *ex vivo* tissues have been an exploratory approach before the eventual *in vivo* applications. In this exploratory study, patients with histopathologic evidence malignancy involving the free border of aryepiglottic fold were selected and compared with the other normal subsite. The findings of the study are discussed in the present paper. 

## 2. Materials and Methods

In total, twenty tissue samples were studied comparing the malignant tissue site with the corresponding normal site in each patient from January 2007 to December 2007. Ten patients with age range 43 years to 75 years and male to female ratio of 9 : 1 were considered for biopsy. Patients with unilateral marginal zone or the aryepiglottic fold malignancy were chosen because it is a transition area from laryngeal mucosa to hypopharyngeal mucosa. This is also known as “laryngopharynx” and is representative of the upper aerodigestive tract histologically. The marginal zone on the other side was grossly free of lesion as it appeared as soft and supple tissue. This study was approved for one year by the Manipal University Ethical Clearance Committee.

**Figure 1:**
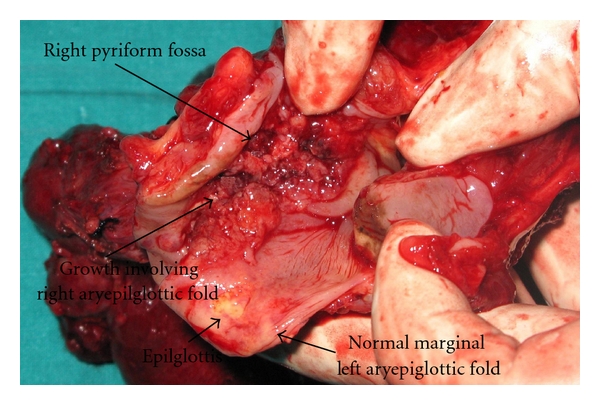
Pictorial presentation of gross laryngeal specimen after excision showing marginal zone formed by the upper margin of the aryepiglottic fold (AEF). The right AEF (big spot) showing ulceroproliferative growth and left AEF (small spot) that appears normal are indicated.

The biopsy specimens were taken from the growth and the corresponding normal side (Figure 1). These were put in individual saline bottles and delivered to the laser spectroscopy department. All the specimens were snap frozen in liquid nitrogen and passively thawed before subjected to Raman studies. A total of twenty samples were subjected to this study. A mirror image of all biopsy specimens were also sent for confirmative histopathology. Histopathologically, the ten malignant specimens were diagnosed to be squamous cell carcinoma (six patients had moderately differentiated while two patients each had infiltrating type and poorly differentiated carcinoma).

## 3. Laser Raman Spectroscopy

**Figure 2:**
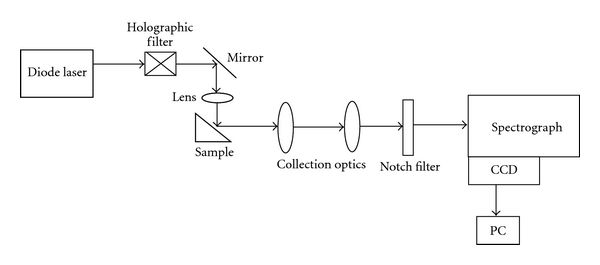
Schematic of the Raman instrumentation.

Raman spectra were recorded using the setup which was assembled by us [14, 15, 20, 22, 24, 31, 33]. In brief, this instrumentation employed diode laser (SDL-8530 785 nm, 100 mW) for excitation and HR 320 spectrograph (600 g/mm blazed at 900 nm) and spectrum one liquid N_2_-cooled CCD for dispersion and detection of Raman signals. The Rayleigh scattering was filtered out using holographic filter (HSBF-785.0; Kaiser Optics). A schematic of the Raman instrumentation is presented in Figure 2. More than six spectra were recorded in each tissue. Each spectrum was acquired for 30 seconds and averaged over 20 accumulations. These experimental settings were kept constant during the study. Samples were kept moist in saline during spectral acquisition. The recorded spectra were postcalibrated with a cubic fit to known frequencies of Tylenol.

## 4. Data Analysis

**Figure 3:**
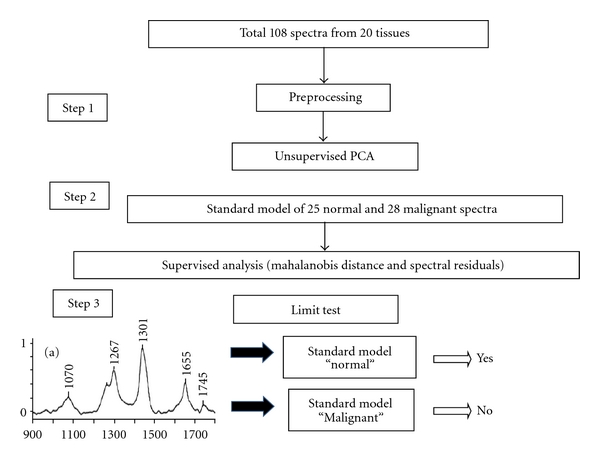
Flow chart of the study design.

The spectra were baseline corrected, smoothened, calibrated using diode adjust algorithms in Grams 32 (Galactic Industries corporation, USA) [35] and normalized over *δ*CH_2_ band. The preprocessed spectra were then subjected to Principle Component Analysis (PCA), a known data reduction technique where huge spectral data are decomposed into small independent variables known as “factors” and contributions of these factors were called “scores.” Spectral data Analysis was carried out over entire region as well as several selected short regions besides derivatives of the same regions for standardization purposes. Total percentage variance, eigenvalues, and factor profiles were employed for standardization of PCA. Trail runs were carried out using 20, 15, 12, and 9 factors. In our analysis spectral range of 900–1750 cm^−1^ with 9 factors gave optimum results. Further data analysis was carried out under these conditions. Analysis was carried out in unsupervised and supervised modes. In the unsupervised approach, scores of factor were used as discriminating parameter whereas, in the supervised mode, Mahalanobis Distance and spectral residuals were used as discriminating parameters [35, 36]. We have also explored match/mismatch “limit test” approach which is known to bring out objective and unambiguous discrimination [14, 15, 20, 22, 24, 31, 33]. The flow chart of the study design is shown in Figure 3.

## 5. Results and Discussion

**Figure 4:**
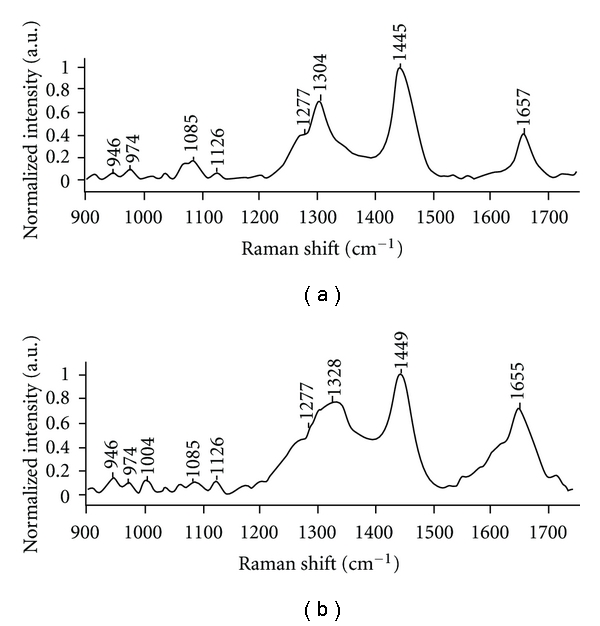
Mean spectra of (a) normal and (b) malignant hypopharyngeal tissues.

Mean Raman spectra of normal and malignant hypopharyngeal tissues are shown in Figure 4. On cursory examination, mean normal spectrum exhibits weak 1650 cm^−1^, *δ*CH_2_ band at around 1445 cm^−1^, sharp peaks at 1304 and 1277 cm^−1^, and a broad peak at 1085 cm^−1^. These spectral features indicate abundance of lipids in normal hypopharyngeal tissues. On the other hand, mean malignant spectrum, distinguished by broad and strong amide I at around 1655 cm^−1^, red shifted *δ*CH_2_ at around 1449 cm^−1^, broad amide III, and sharp peak at 1004 cm^−1^ suggest increased protein content with respect to normal tissues. We have observed similar features of abundance of lipids and proteins in normal and malignant oral tissue spectra, respectively [14–17]. 

**Figure 5:**
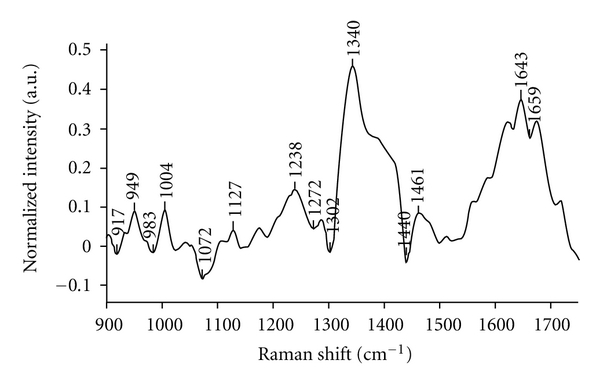
Difference spectrum of mean malignant minus mean normal spectrum.

**Figure 6:**
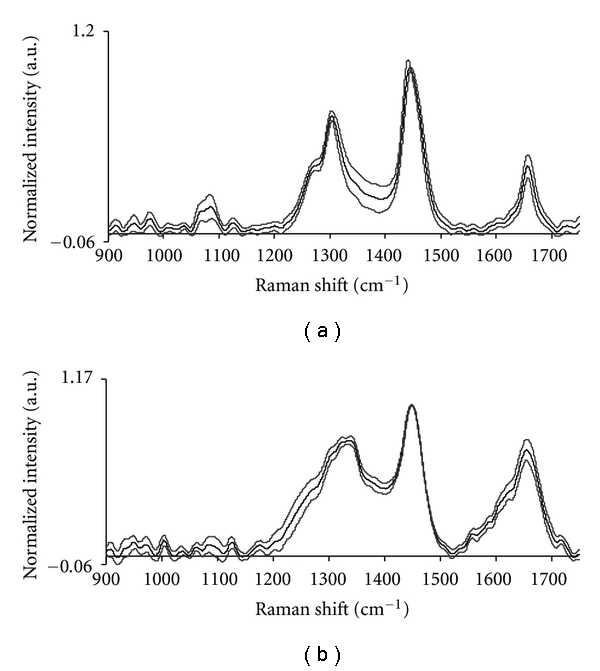
Mean and standard deviation spectra of hypopharyngeal tissues. (a) Normal. (b) Malignant.

For better correlation of spectral and biochemistry, the difference spectrum was computed by subtracting mean normal spectrum from mean malignant spectrum as shown in Figure 5. All the negative peaks (917, 983, 1072, 1302, 1440 cm^−1^) seen in Figure 5 were contributed by normal spectrum attributable to lipids whereas all positive peaks (949, 1004, 1127, 1238, 1340, 1643 cm^−1^) were from malignant spectrum which could be assigned to proteins. Besides high protein content, spectral features of the mean malignant tissue spectrum also indicate the presence of additional biomolecules like DNA (1340 cm^−1^) and variations in secondary structure of the protein as indicated by amide I and III bands [37, 38]. We have also verified heterogeneity of spectra among same class of tissues, for example, normal and malignant tissues, by computing mean and standard deviation of normal as well as malignant spectra as illustrated in Figure 6. In Figure 6, the mean and standard deviation spectra indicate very minor heterogeneity and minor intensity differences. 

It is well known that there are several multivariate statistical methods available for the spectroscopist for data mining. We have opted PCA for spectral data analysis in order to discriminate malignant from normal tissue types. In our method of PCA, the mean of all samples in the data set is first formed. The differences of this mean from each sample are calculated to give the variations of each sample from the mean. With n samples, each having p data points, we thus get an [n × p] matrix of these variations. Because all the samples contain more or less the same components (e.g., lipids, proteins, and collagen) the large amount of data can be represented by a much smaller set of components and their contributions to each spectrum depending on their concentrations. In matrix language this implies that the [n × p] matrix of variations discussed above is highly redundant. It will have only a few eigenvectors (principal components), and the eigenvalues of these will rapidly come down to almost zero after the first few. Solving the eigen value-eigen vector problem will give us the principal components (factors), % variance (contribution of the factors to the variations in the data set), and scores of factors for each sample. The scores for a given sample correspond to the contribution of each principal component to the variation of that sample from the mean. It is therefore possible to simulate the observed spectrum of any sample by multiplying the eigen vectors with their respective scores for that sample and adding these products to the mean of the data set.

As described above, in PCA large amount of spectral data is expressed by independent variables called eigenvectors, factors, or principal components and their scaling constants, scores. Scores of factors are often used as parameters to achieve objective discrimination. As mentioned earlier in Section 4, analysis was carried out in two different approaches: (1) unsupervised analysis, (2) supervised analysis. We have successfully tested these approaches in our earlier Raman spectroscopic studies of cervix, oral, and breast cancers [14, 15, 20, 22, 24, 31, 33]. 

**Figure 7:**
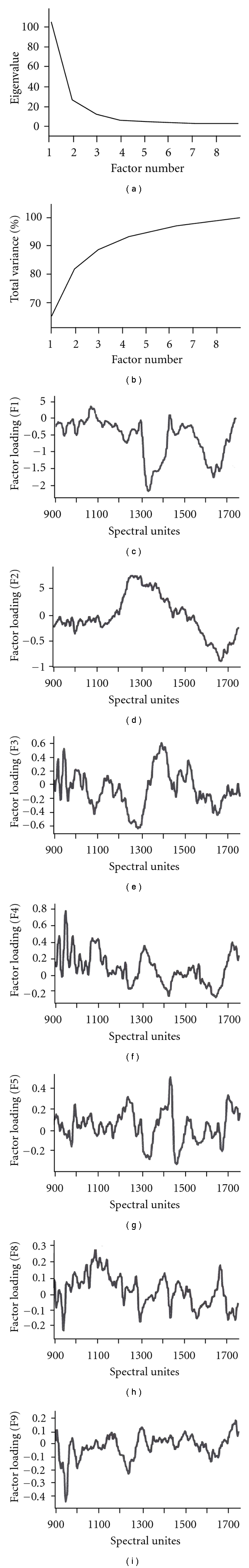
PCA of normal and malignant larynx tissue (a) eigen value, (b) total % variance, (c) loadings of factor 1, (d) loadings of factor 2, (e) loadings of factor 3, (f) loadings of factor 4, (g) loadings of factor 5, (h) loadings of factor 8, (I) loadings of factor 9.

**Figure 8:**
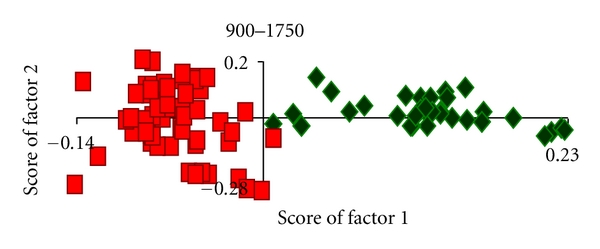
Unsupervised analysis of hyphoharyngeal spectra. *◊* Normal. ■ Malignant.

In the first approach a total of 108 spectra from normal and malignant hypopharyngeal tissue were ascertained for unsupervised classification. Profiles of the factor loadings are shown in Figure 7. The first five factors contribute 94% of variance, and the last two account for noise. There is clustering of normal and malignant spectra based on score of factor 1, as shown in Figure 8. The scores of factor 1 for normal spectra were generally positive for malignant and negative for normal tissues with a mean standard deviation of 0.011 ± 0.05 and −  0.06 ± 0.03, respectively. Mean and standard deviation values of normal and malignant spectra of score of factor 2 were 0.02 ± 0.05 and −  0.01 ± 0.12. A minor overlap is present between clusters up to ±1 standard deviation, which indicates a sensitivity and specificity of 75%. 

Analysis by score of factors may give a clear classification of tissues for discrimination; however this approach of classification is somewhat cumbersome and tedious because diagnosis of a sample needs entire analysis to be repeated along with new spectra. Moreover, it may be of limited practical utility for the end-users, clinicians, since a visual decision-making is involved in the case of borderline samples. In view of these considerations, we have developed a second method using multiple discriminating parameters to give a better and objective diagnosis. For this, like in any analytical technique where standards with calibration curves are used for routine analysis, spectra of a set of clinically/pathologically diagnosed samples can be used as a *standard calibration set*. This standard calibration set can be subjected to PCA to derive parameters which will be highly characteristic for any sample of that type. Any *test sample* can then be included in the set, and the corresponding parameters for the test sample can be compared to the mean parameters for the set to decide whether the test sample belongs to that set and, if so, with what statistical probability. We have thus several statistical parameters available for decision-making in PCA, especially when standard calibration sets are used. In this mode besides scores of factor, PCA provides other discriminating parameters of classification such as Mahalanobis distance [35, 36] (a measure of proximity of two spectra) and spectral residuals (squared error sum of difference between recorded and simulated spectrum). Hence the supervised mode which provides multiple discriminating parameters is better suited for objective diagnosis by spectroscopy methods, especially for clinical conditions. In this analysis certain certified samples were used to develop standard sets. A given spectra was compared with these sets to decide whether it belongs to the standard set with the statistical probability of inclusion. If Mahalanobis distance of the test spectra has values more than three, compared to the training sets it had a probability of 0.5% or less of being grouped as the same class. The Mahalanobis distance [35] is normally expressed in units of standard deviation and expressed as

(1) $$D^{2}\;=\;\left(S_{\text{test}}\right)M^{-1}\left(S_{\text{test}}\right)'.$$

In the previous equation, *S*_test_ is the vector of the scores and the sum of squared spectral residuals for a given test sample, where

(2) $$M\;=\;S^{\prime }S/\left(n-1\right).$$

“*S*” contains the corresponding parameters for the calibration set (*n *standards).

**Figure 9:**
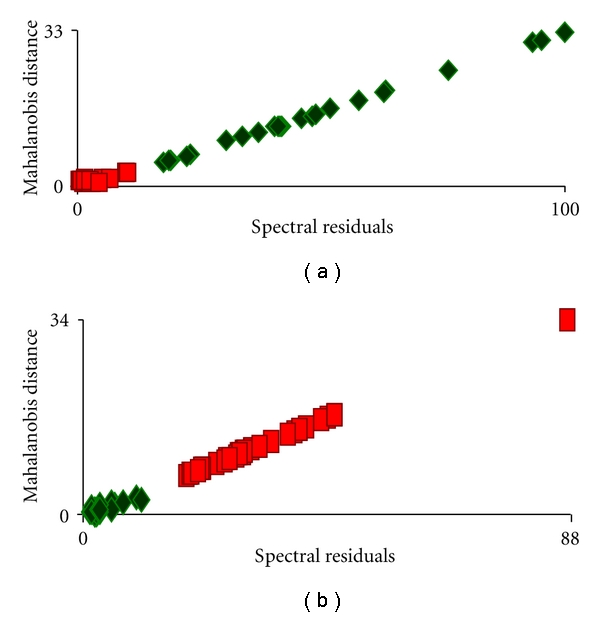
Supervised analysis of hyphoharyngeal tissue spectra. *◊* Normal. ■ Malignant. (a) Verification of the standard sets against malignant standard set. (b) Evaluation of the standard sets against normal standard set.

In our study, we have selected 25 normal and 28 malignant spectra randomly based on a score of factor 1 and histopathological certification. The consistency of the standard sets was verified by rotating spectra from training sets and comparing them against both training sets. The spectra corresponding to same class of training sets procure lower Mahalanobis distance and spectral residues and vice versa. As an example, results obtained against a malignant training set were shown in Figure 9(a). The mean Mahalanobis distance of normal and malignant spectra were 15.1 ± 8.13 and 0.93 ± 0.61, respectively. The mean spectral residual values for normal and malignant tissues were 48.11 ± 24.23 and 3.52 ± 2.99, respectively. 

These standard sets were further evaluated by spectra that were not involved in training sets wherein the test spectra were compared against both the training sets. A good discrimination was achieved, for example, as shown in Figure 9(b) of results obtained against the normal training set. The mean Mahalanobis distance of normal and malignant were 3.37 ± 2.47 and 32.05 ± 12.8, respectively.

The approach of computing mahalanobis distance and spectral residuals is further extended to multiparametric “limit test” approach in order to achieve objective and unambiguous discrimination. This is a typical match/mismatch approach against a standard set. A given spectra was compared with fixed values of inclusion/exclusion criteria for analysis of Mahalanobis distance, spectral residuals, and scores of factors. Based on these values of a given spectrum being within or without the set limits, the spectrum was labeled as “Yes/possible/pass (match)” or “No/fail (mismatch)” respectively. In this analysis, as an example, a normal spectrum should show “Yes/possible/pass” when compared to a normal standard set and “No/fail” with other standard sets and vice versa. Since the spectra are matched against all the standard sets, a reasonable and objective discrimination is achieved before concluding the type of the tissue. All malignant and nonmalignant spectra show “match” and “no match”, respectively, when compared with a malignant standard set (Table 1). In this table, spectra 1–48 were normal tissue spectra, and spectra 49–108 were of malignant tissues. Efficacy of this approach was demonstrated in our earlier Raman studies of oral, breast, cervix, stomach, and colon cancers [14, 15, 20, 22, 24, 31, 33].

The results obtained in this pilot study provide reliable evidence on Raman spectroscopic discrimination of malignant hypopharyngeal tissues from normal. The limit test approach is significant in early clinical diagnosis as a clinician or technician can match a recorded spectrum with the training sets once they are developed for different pathological conditions aiding easy objective decisions, which is the ground stone for attempting curative treatment plan. 

The future lies in designing a fibre probe tissue interface obtaining calibrated intensity information and depth ranging information. Raman probes may be designed to eliminate scattering distortion while providing the endoscopic images of the chemical and/or morphological properties of the tissue to complement tissue diagnosis on immediate basis during surgery or a diagnostic procedure.

## 6. Conclusion

Tobacco chewing and smoking is rampant and hazardous in an already rapidly increasing population. This doubles the need and effort to make early, easy, and immediate detection of malignant changes of the abused and vulnerable hypopharyngeal tissues. Though there are various methods to detect cancerous tissue, each has a drawback that may be overcome by expanded study of an alternative modality of tissue diagnosis such as conventional Raman spectroscopy. Spectral signatures were characterized by variations in the protein and lipid content at biomolecular level. Discriminating parameters scores of factor, Mahalanobis distance, spectral residuals provided clear classification between normal and malignant tissue types. Further the “limit test” approach also provided unambiguous and objective discrimination, which is more user-friendly and adaptable to routine clinical practice as it requires a minimally trained person and even a clinician and technician can come to a conclusion before taking a decision.

However, a confirmed application of Raman spectroscopy technique will come to force following prolonged prospective study and introducing endoscopy friendly Raman probes.

## Figures and Tables

**Table 1:** Limit test approach against normal standard set (1–48 normal, 49–108 malignant).

Sample Number	Match	Limit test
1	POSSIBLE	PASS (PP?#)
2	POSSIBLE	PASS (PP?#)
3	YES	PASS (PPP#)
4	YES	PASS (PPP#)
5	YES	PASS (PPP#)
6	POSSIBLE	PASS (PP?#)
7	YES	PASS (PPP#)
8	YES	PASS (PPP#)
9	YES	PASS (PPP#)
10	YES	PASS (PPP#)
11	YES	PASS (PPP#)
12	YES	PASS (PPP#)
13	POSSIBLE	PASS (PP?#)
14	POSSIBLE	PASS (PP?#)
15	YES	PASS (PPP#)
16	YES	PASS (PPP#)
17	YES	PASS (PPP#)
18	POSSIBLE	PASS (PP?#)
19	YES	PASS (PPP#)
20	YES	PASS (PPP#)
21	YES	PASS (PPP#)
22	YES	PASS (PPP#)
23	POSSIBLE	PASS (PP?#)
24	YES	PASS (PPP#)
25	POSSIBLE	PASS (PP?#)
26	YES	PASS (PPP#)
27	POSSIBLE	PASS (PP?#)
28	YES	PASS (PPP#)
29	POSSIBLE	PASS (PP?#)
30	YES	PASS (PPP#)
31	YES	PASS (PPP#)
32	POSSIBLE	PASS (PP?#)
33	YES	PASS (PPP#)
34	YES	PASS (PPP#)
35	YES	PASS (PPP#)
36	YES	PASS (PPP#)
37	YES	PASS (PPP#)
38	YES	PASS (PPP#)
39	POSSIBLE	PASS (PP?#)
40	POSSIBLE	PASS (PP?#)
41	YES	PASS (PPP#)
42	POSSIBLE	PASS (PP?#)
43	YES	PASS (PPP#)
44	YES	PASS (PPP#)
45	YES	PASS (PPP#)
46	YES	PASS (PPP#)
47	POSSIBLE	PASS (PP?#)
48	POSSIBLE	PASS (PP?#)
49	NO	FAIL (FFF#)
50	NO	FAIL (FFF#)
51	NO	FAIL (FFF#)
52	NO	FAIL (FFF#)
53	NO	FAIL (FFF#)
54	NO	FAIL (FFF#)
55	NO	FAIL (FFF#)
56	NO	FAIL (FFF#)
57	NO	FAIL (FFF#)
58	NO	FAIL (FFF#)
59	NO	FAIL (FFF#)
60	NO	FAIL (FFF#)
61	NO	FAIL (FFF#)
62	NO	FAIL (FFF#)
63	NO	FAIL (FFF#)
64	NO	FAIL (FFF#)
65	NO	FAIL (FFF#)
66	NO	FAIL (FFF#)
67	NO	FAIL (FFF#)
68	NO	FAIL (FFF#)
69	NO	FAIL (FFF#)
70	NO	FAIL (FFF#)
71	NO	FAIL (FFF#)
72	NO	FAIL (FFF#)
73	NO	FAIL (FFF#)
74	NO	FAIL (F?F#)
75	NO	FAIL (FFF#)
76	NO	FAIL (FFF#)
77	NO	FAIL (FFF#)
78	NO	FAIL (FFF#)
79	NO	FAIL (FFF#)
80	NO	FAIL (PFF#)
81	NO	FAIL (PFF#)
82	NO	FAIL (FFF#)
83	NO	FAIL (FFF#)
84	NO	FAIL (FFF#)
85	NO	FAIL (FFF#)
86	NO	FAIL (FFF#)
87	NO	FAIL (F?F#)
88	NO	FAIL (FFF#)
89	NO	FAIL (FFF#)
90	NO	FAIL (FFF#)
91	NO	FAIL (P?F#)
92	NO	FAIL (P?F#)
93	NO	FAIL (FFF#)
94	NO	FAIL (FFF#)
95	NO	FAIL (FFF#)
96	NO	FAIL (FFF#)
97	NO	FAIL (FFF#)
98	NO	FAIL (F?F#)
99	NO	FAIL (FFF#)
100	NO	FAIL (FFF#)
101	NO	FAIL (FFF#)
102	NO	FAIL (FFF#)
103	NO	FAIL (FFF#)
104	NO	FAIL (FFF#)
105	NO	FAIL (FFF#)
106	NO	FAIL (FFF#)
107	NO	FAIL (PFF#)
108	NO	FAIL (FFF#)
